# Synthetic pathways and processes for effective production of 5-hydroxytryptophan and serotonin from glucose in Escherichia coli

**DOI:** 10.1186/s13036-018-0094-7

**Published:** 2018-03-15

**Authors:** José-Aníbal Mora-Villalobos, An-Ping Zeng

**Affiliations:** 1 Institute of Bioprocess and Biosystems Engineering, Hamburg University of Technology, Hamburg, Germany; 2 Centro Nacional de Innovaciones Biotecnológicas, Centro Nacional de Alta Tecnología, San José, Costa Rica

**Keywords:** 5-hydroxytryptophan, Serotonin, Protein engineering, Aromatic amino acid hydroxylase, Synthetic pathway

## Abstract

**Background:**

Tryptophan derivatives such as 5-hydroxytryptophan (5HTP) and serotonin are valuable molecules with pharmaceutical interest. 5HTP is presently mainly obtained by extraction from the plant *Griffonia simplicifolia* and serotonin is produced by chemical synthesis. A simple biotechnological method for the production of these compounds is desired.

**Results:**

In a first attempt to synthesize serotonin from glucose, we used a single engineered *Escherichia coli* strain and observed a low production of maximal 0.8 ± 0.2 mg/L of serotonin, probably due to the undesired site-reaction of direct decarboxylation of tryptophan and the consequent decrease of the precursor 5HTP. To circumvent this problem, we have constructed a stepwise system in which the 5HTP production and the serotonin conversion are separated. 962 ± 58 mg/L of 5HTP was produced in the first step using a recombinant strain with a semi-rationally engineered aromatic amino acid hydroxylase, the highest concentration reported so far. In a subsequent step of 5HTP bioconversion using a recombinant strain harboring a tryptophan decarboxylase, 154.3 ± 14.3 mg/L of serotonin was produced.

**Conclusions:**

We present results of a two-stage fermentation process for the production of 5HTP and serotonin. The first strain is a highly efficient 5HTP producer, and after fermentation the supernatant is separated and used for the production of serotonin. This is the first report for the microbial production of serotonin from glucose.

**Electronic supplementary material:**

The online version of this article (10.1186/s13036-018-0094-7) contains supplementary material, which is available to authorized users.

## Background

Tryptophan is an essential amino acid with medical, industrial and pharmaceutical importance. Potential therapeutic agents have stimulated the interest in the design and synthesis of tryptophan-related structures, which could have direct health benefits or may work as key biosynthetic precursors for other molecules. 5-Hydroxytryptophan (5HTP) and serotonin are two important tryptophan derivatives. 5HTP is an intermediate molecule in the biosynthesis of serotonin, and over the last 30 years, it has been used to treat a wide variety of conditions related to serotonin imbalance, such as depression, insomnia, fibromyalgia, chronic headaches and binge eating associated with obesity [1]. For many years there have been chemical synthesis methods reported [2, 3], still its production is not economically feasible, and the main supply depends on the extraction from seeds of the African plant *Griffonia simplicifolia*. Serotonin is naturally present in animals and plants, it is produced by the decarboxylation of 5HTP or the hydroxylation of tryptamine, respectively. In both cases, it is implicated in fundamental physiological roles (Kang et al., [4]; Turner et al., [5]). Analogs that resemble serotonin structure act on a wide range of therapeutic targets, such as phosphodiesterase, 5-hydroxytryptamine receptors, cannabinoid receptors and HMG-CoA reductases. Many of these targets contain a binding pocket that recognizes the indole scaffold [6].

The indole aromatic heterocyclic backbone, present in 5HTP and serotonin, is a valuable molecular framework that provides a plethora of opportunities for medical chemistry and drug discovery. It is not unusual that the structure of drugs, or their precursors, resemble bioactive molecules with the elimination, addition or modification of functional groups. Therefore, 5HTP and serotonin, could serve as building blocks for active ingredients that are used as pharmaceuticals, such as melatonin (sleep cycle regulator) [7], triptans (migraines) [8], β-carbolines (sedative, anticonvulsant, antitumor, antimicrobial) [9] and eudistomins (antiviral) [10], among many others. Furthermore, 5HTP and serotonin *per se* possess free radical scavenging and antioxidant activity [11, 12]. Biotechnology itself presents as a tempting promise for the production of these molecules with high yields, in short time with low costs [13].

**Fig. 1:**
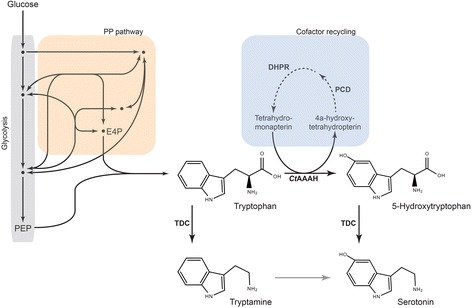
Novel artificial pathway for the biosynthesis of 5-hydroxytryptophan and serotonin in *E. coli.* PEP, phosphoenolpyruvate; E4P, erythrose 4-phosphate; *Ct*AAAH, aromatic amino acid hydroxylase from *Cupriavidus taiwanensis*; TDC, tryptophan decarboxylase from *Catharanthus roseus*; PCD, pterin-4 alpha-carbinolamine dehydratase from human; DHPR, dihydropteridine reductase from human

For several decades, different approaches have been adopted to channel the carbon flux towards the production of aromatic amino acids. Current developed metabolically engineered strains can produce tryptophan with high titer and yield [14, 15]; they are effective platforms for the microbial production of tryptophan derivatives. Two enzymes are involved in the further conversion of tryptophan to 5HTP and serotonin: tryptophan hydroxylase (TPH) and aromatic amino acid decarboxylase (Fig. 1). Tryptophan hydroxylase has been expressed in *Escherichia coli* with a low enzymatic activity, the solubility and stability of the enzyme seem to be affected when expressed in *E. coli* [16, 17]. Different groups have reported the engineering of a prokaryotic phenylalanine hydroxylase in order to change the substrate preference to tryptophan [18–20]. Still, the production of 5HTP is compromised by the activity of the enzyme. In this case, further rounds of optimization are necessary in order to achieve a higher productivity. Regarding the decarboxylation, Noé et al. [21] reported that both, tryptophan and 5HTP, are natural substrates of tryptophan decarboxylase (TDC) from *Catharanthus roseus.* This same enzyme has been used for the production of serotonin in *E. coli*. However 5HTP was supplied as a substrate, and the yield was quite low (35 mg/L) [22].

Directed evolution by saturation mutagenesis (SM) has proven to be a useful method for protein engineering in a variety of different applications. The access of tertiary structures and the development of in silico screening and prediction methods are helpful tools in this endeavor. Protein evolution methods often suffer from bottlenecks in the design of the library and screening process due to the high number of theoretical combinations of mutants. Iterative saturation mutagenesis (ISM) is an option to overcome some of the problems, especially when it is coupled with smart-libraries that integrate structural and evolutionary data, the combination of both reduces drastically the screening efforts required to select novel enzymes with the desired activity [23–25].

Here, we engineered an artificial pathway in *E. coli* for the production of 5HTP and serotonin from a simple carbon source. We extended the tryptophan pathway using an engineered phenylalanine hydroxylase from *Cupriavidus taiwanensis* (*Ct*AAAH) and an endogenous cofactor with an artificial regeneration system for the production of 5HTP. In a second step, 5HTP was converted into serotonin with a strain transformed with a tryptophan decarboxylase enzyme.

## Results

### Conceptual design of the pathway for the production of 5HTP and serotonin

There are two possible pathways to produce serotonin from tryptophan. Tryptophan can be hydroxylated into 5HTP and further converted into serotonin; alternatively, if tryptophan is decarboxylated to tryptamine, this can be then converted into serotonin. In the case of the second pathway mentioned above, Park et al. [26] observed a low tryptamine-5-hydroxylase (T5H) activity in *E. coli*: 0.15 mM of serotonin was produced when tryptamine was added to the media with concentration rounded 0.5 mM, and serotonin concentration did not increase when the substrate concentration increased.

Previously, we engineered an aromatic amino acid hydroxylase from *C. taiwanensis* (*Ct*AAAH-W192F, from here on referred as *Ct*AAAH-F) with which 2.5 mM of 5HTP was produced in *E. coli* in media supplied with 5 mM of tryptophan [20]. Furthermore, both, tryptophan and 5HTP, are natural substrates of TDC [21, 26]. Hence, we chose to produce serotonin via 5HTP from a simple carbon source glucose.

### Construction of a strain for the production of 5HTP from glucose

A pterin (cofactor) reconstitution pathway was incorporated, via plasmid (Pl) or via genome integration (Gi), in the tryptophan producer strain S028 [14], whereby strains TrpD-Pl and TrpD-Gi were derived. No differences regarding growth and tryptophan production were observed between the original strain and the newly generated strains (Fig. 1. Additional file 1).

**Fig. 2:**
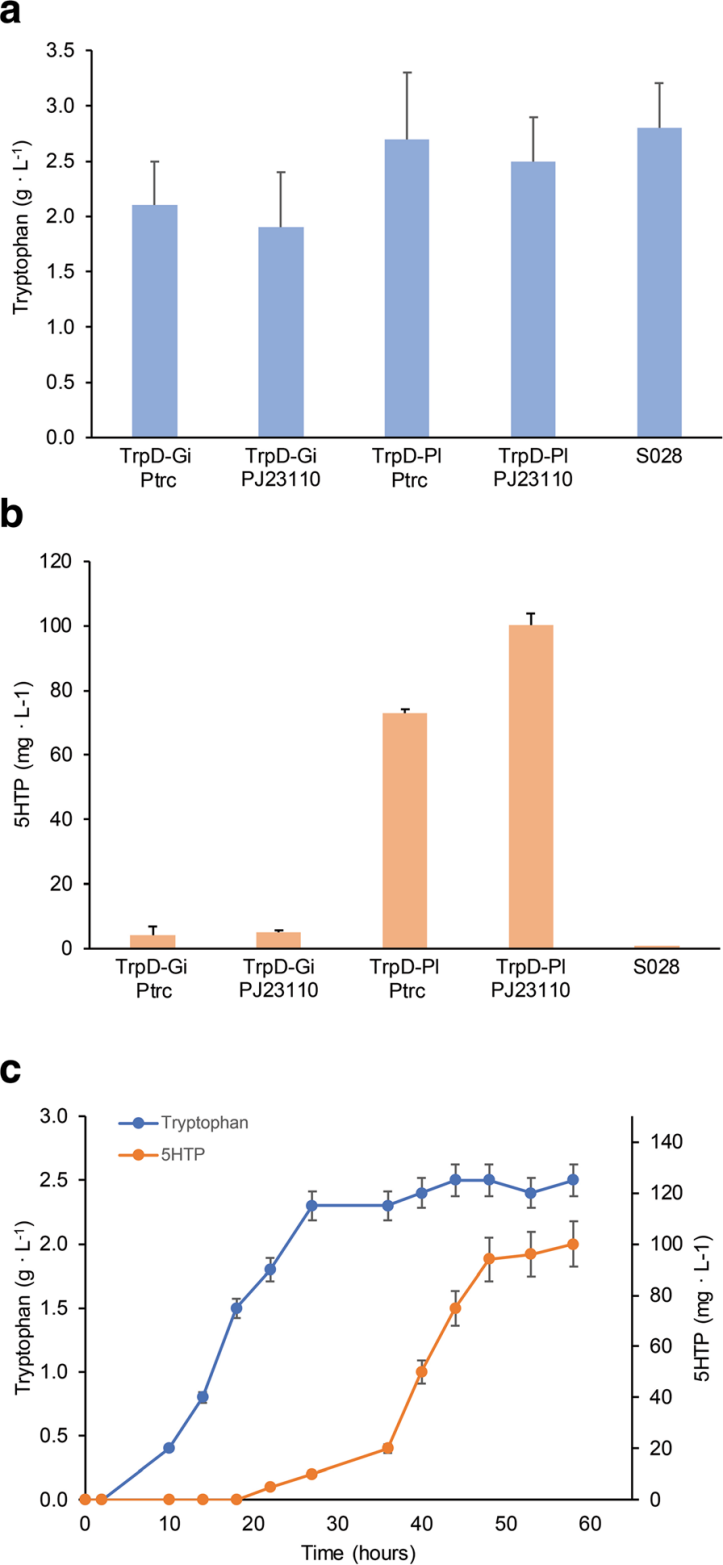
5HTP production in different *E. coli* strains. **a** tryptophan production and (**b**) 5HTP production of TrpD-Gi and TrpD-Pl strains carrying the *Ct*AAAH-F gene under control of the P_trc_ and P_J23110_ promoters respectively. **c** Tryptophan and 5HTP production of TrpD-Pl / pACJ23-*Ct*AAAH-F over time

These strains were transformed with two variants of the pACYCDuet-*Ct*AAAH-F. The strong-inducible T7 promoter from this plasmid was exchanged by a strong-constitutive promoter (P_trc_) and by a medium strength-constitutive promoter (P_J23110_), creating pACPtrc-*Ct*AAAH-F and pACPJ23-*Ct*AAAH-F, respectively. In general, a slight reduction in tryptophan production was observed in the TrpD-Gi strain when transformed with the mentioned plasmids. Moreover, a lower amount of 5HTP was detected in the culture of TrpD-Gi compared with TrpD-Pl. With TrpD-Pl, the strain carrying pACJ23-*Ct*AAAH-F produced over 2.5 g/L of tryptophan and 100 mg/L of 5HTP from glucose in shaken flasks after 60 h fermentation. The strain with pACPtrc-*Ct*AAAH-F produced a similar amount of tryptophan, but around 25% less 5HTP (Fig. 2a-b).

Although TrpD-Pl / pACJ23-*Ct*AAAH-F produced the highest amount of 5HTP within the set of analyzed strains, still there is a high amount of residual tryptophan (2.7 ± 0.6 g/L) when compared with the target product (5HTP: 100.4 ± 3.5 mg/L), also the 5HTP level did not increase with the tryptophan concentration level. Tryptophan production reached a steady state 24 h after the inoculation, meanwhile 5HTP production was stable after 48 h (Fig. 2c). For this reason, we decided to further engineer the *Ct*AAAH-F enzyme using the residual tryptophan as the selection criteria for the protein evolution.

### Optimization of the 5HTP pathway via protein engineering of *Ct*AAAH

**Fig. 3:**
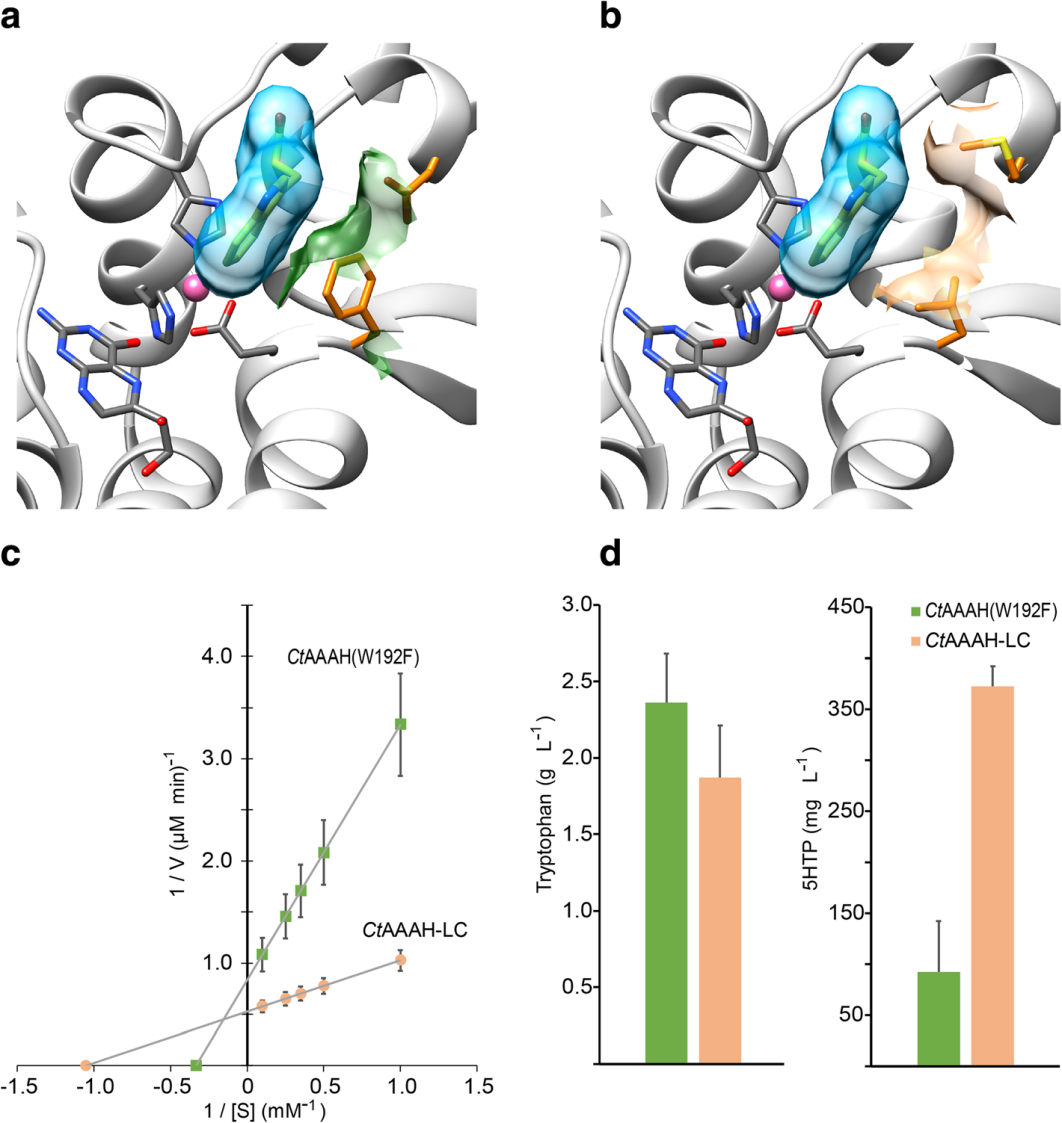
Protein engineering of *Ct*AAAH-F. **a*** Ct*AAAH-F binding pocket predicted: the atoms of the cofactor and 2-His-1-caboxylate facial triad are present in gray, iron atom in pink; tryptophan surface is shown in blue, F197 and E219 atoms are present in orange and their surfaces are shown in green. **b*** Ct*AAAH-LC binding pocket predicted: as in a., but L197 and C219 are present in orange, as well as their surfaces. **c** Lineweaver-Burk plot of *Ct*AAAH-F and *Ct*AAAH-LC. **d** tryptophan and 5HTP production after batch fermentation. Values in the graph are the average of triplicates, error bars correspond to the standard error of the mean (SEM)

Two positions of the *Ct*AAAH-F were subjected to SM using the reduce codon strategy proposed by Kille et al. [24]. Residues Phe197 and Glu219 were selected for SM because these positions are part of the binding pocket of the enzyme in the region that interacts with the aromatic ring of the substrate and they are also near to the cofactor. These residues also play an important role in defining the pocket’s shape and volume (Fig. 3a).

We amplified part of the gene and vector backbone from pACPJ23-*Ct*AAAH-F using phosphorothioate primers; after cleavage with an I_2_/EtOH solution we generated a fragment with sticky ends. Four synthetic oligos which include positions Phe197 and Glu219 were used to generate two independent libraries. Hybridized fragments were transformed into the strain BL21(DE3) *ΔtnaA* carrying plasmid pSenTrp(-LVA).2 which encodes for an intracellular tryptophan sensor. Enzymes with low activity should not consume tryptophan, consequently fluorescence should be high; and vice versa, a low fluorescence means low intracellular tryptophan concentration due to the activity of the enzyme that converted it into 5HTP.

In order to investigate the quality of the libraries, the *Ct*AAAH-F gene of 48 colonies was sequenced: 43 colonies were identical to the original sequence except for the saturated site, four sequences presented indels near the sticky ends where the molecules hybridized and one sequence presented a point mutation out of the two categories just mentioned.

A total of 1673 colonies were screened in M9 media supplied with 1 mM L-tryptophan, 823 colonies from the Phe197-library (F197-Lib) and 850 from the Glu219-library (E219-Lib). We selected 167 colonies from the F197-L and 124 from the E219-L with low or no fluorescence, and transferred them to a new M9-plate supplied with 2 mM L-tryptophan. Successive steps of 1 mM tryptophan increases were done until no evident change in fluorescence was detectable (compared to the previous plate). At the end, four and three single colonies with low fluorescence from F197-Lib and E219-Lib were identified in the 5 mM tryptophan plates.

DNA from these colonies was extracted and the *Ct*AAAH-F gene was completely sequenced. Out of the F197-Lib, one colony had the same genotype as the wild-type (further analysis revealed a mutation in the tryptophan sensor), two had a mutation that substituted phenylalanine for leucine (F197 L) and one sequence had an isoleucine in position 197 (F197I). All sequences obtained from the E219-Lib presented a cysteine in amino acid position 219 instead of glutamate (E219C).

Enzymatic assays with the three identified variants were done and compared with *Ct*AAAH-F, kinetic parameters are listed in Table 1. Leu197 and Cys219, performed better in the tryptophan hydroxylation assay when compared with the *Ct*AAAH-F. Then, we created the double mutant *Ct*AAAH-F197 L/E219C (*Ct*AAAH-LC) to further explore the combinatorial effect of these residues. The double mutant showed a higher activity than the variants with single mutations. *Ct*AAAH-LC also displayed a lower K_m_ value (0.95 mM) and a higher reaction velocity (V_max_ = 1.9 mM · s^− 1^) when compared to the original *Ct*AAAH-F (Fig. 3c).

**Table 1:** Steady-state kinetic parameters of *Cupriavidus taiwanensis* tryptophan hydroxylase W192F (*Ct*AAAH-F) and variants produced by semi-rational evolution

	K_m_ (mM)	k_cat_ (s^−1^)	V_max_ (mM · s^−1^)	Relative k_cat_/K_m_
*Ct*AAAAH-F	3.0	0.4	1.2	0.13
*Ct*AAAAH-F197I	2.1	1.3	2.7	0.62
*Ct*AAAAH-F197 L	1.6	1.1	1.8	0.69
*Ct*AAAAH-E219C	1.8	1.5	2.7	0.83
*Ct*AAAH-LC	0.95	1.9	1.8	2.00

The production of tryptophan and 5HTP was compared for the TrpD-Pl strains harboring p*Ct*AAAH-F and p*Ct*AAAH-LC plasmids respectively. After 60 h of batch-fermentation, 372.6 ± 19.7 mg/L of 5HTP were produced in the cells carrying the p*Ct*AAAH-LC plasmid, around 3.5 times higher than the cells with p*Ct*AAAH-F. In the case of the cells with p*Ct*AAAH-LC, a reduction in tryptophan production was observed. This optimized strain was used for further serotonin production (Fig. 3d).

### Serotonin production using a single culture

The 5HTP producer (strain TrpD-Pl / p*Ct*AAAH-LC) was transformed with the pCOLAJ23-*TDC*.2 plasmid. After 60 h of fed-batch fermentation the OD_600_ reached a value of 10.2 ± 0.6, and 5.34 ± 0.43 g/L of tryptophan was produced. Final 5HTP production was quite low (7.3 ± 0.6 mg/L) compared with the TrpD-Pl / p*Ct*AAAH-LC (962 ± 58 mg/L) control. Furthermore, no serotonin was produced. We detected 3.03 ± 0.32 g/L of tryptamine in the supernatant, which indicates a strong preference of the TDC enzyme towards tryptophan.

In an attempt to separate the 5HTP and serotonin production stages, TDC was subcloned into the pBAD plasmid; in this case, *TDC* would be induced by arabinose. *TDC* was induced 24 h after inoculation (OD_600_ around 10). We found 1.81 ± 0.29 g/L of tryptamine, similar to the 1.66 ± 0.27 g/L produced in the non-induced control. 21.3 ± 3.5 mg/L 5HTP and 0.8 ± 0.2 mg/L of serotonin were detected in the supernatant after 60 h fermentation.

### Two-step fermentation strategy for the efficient production of serotonin

Decarboxylation by TDC is a key step in the synthetic pathway for serotonin production, and although this enzyme can use both, 5HTP and tryptophan, as a substrate, it has a high preference towards the latter one. Despite the advantages of a single strain production system, it would be difficult to integrate a tight control over the expression of the *TDC*, plus an engineered enzyme with less preference toward tryptophan, but without compromising the 5HTP activity. To circumvent this issue, we decided to separate the final step from the 5HTP production using a two-step culture approach.

**Fig. 4:**
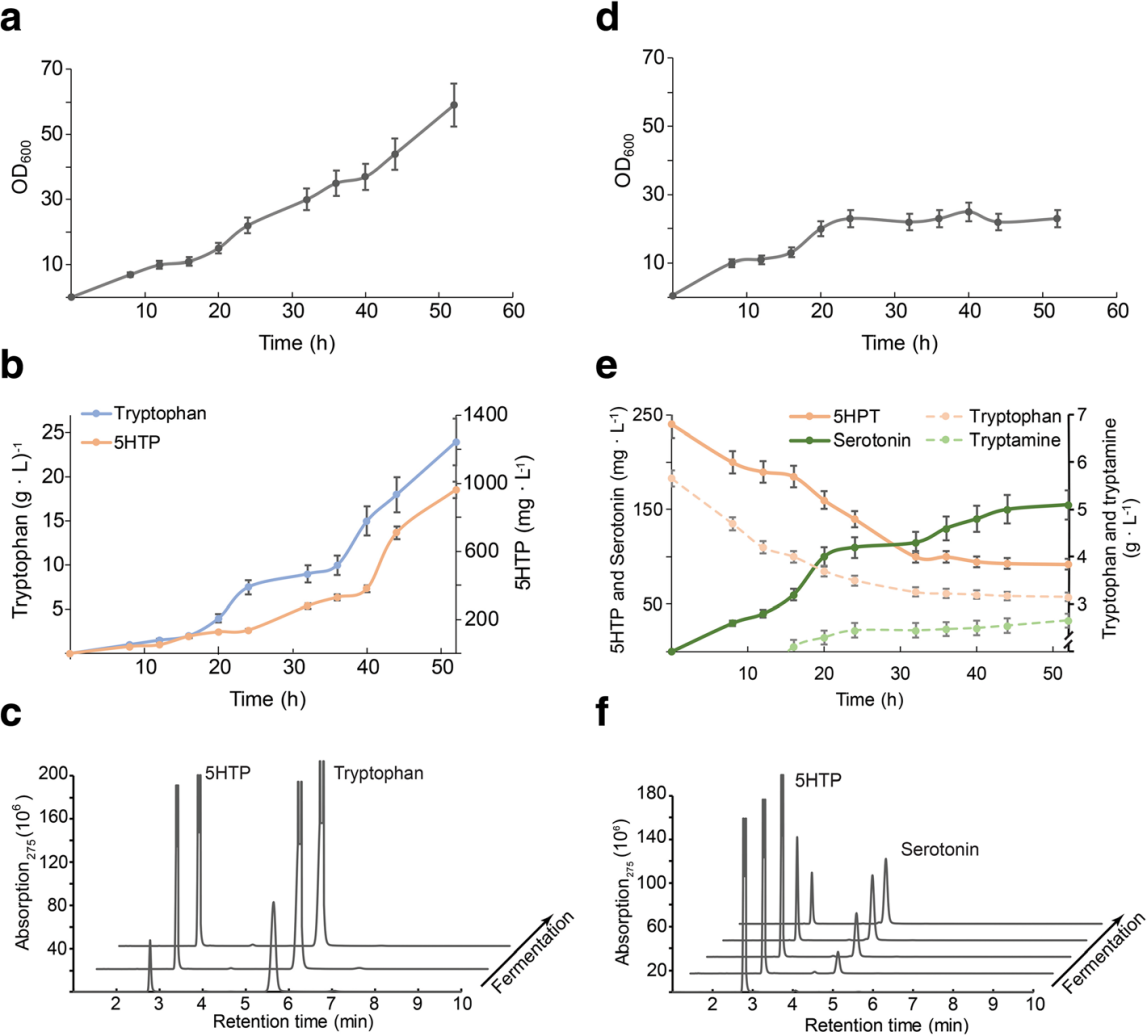
Serotonin production using a two-stage strategy. **a** and **d** growth curves; (**b** and **e**) production/consumption of tryptophan, 5HTP and serotonin. Side reaction (tryptophan conversion to tryptamine) is also indicated in panel e. with dashed lines; **c** and **f** HPLC retention pattern of tryptophan, 5HTP and serotonin; (**a**, **b** and **c**) panels correspond to the TrpD-Pl / pACJ23-*Ct*AAAH-LC fermentation for the production of 5HTP from glucose; panels (**d**, **e** and **f**) correspond to the BL21(DE3) *ΔtnaA* / pCOLAJ23-*TDC* fermentation for the production of serotonin. Values in the graph are the average of triplicates, error bars correspond to the standard error of the mean (SEM)

First, 5HTP was produced from glucose using TrpD-Pl / p*Ct*AAAH-LC with a fed-batch fermentation strategy. After 60 h fermentation, final reached OD was 59 ± 3.1, 23.4 ± 1.4 g/L of tryptophan and 962 ± 58 mg/L 5HTP was produced (Fig. 4a-b). Chromatograms in Fig. 4c show the accumulation of tryptophan and 5HTP, both peaks appear and increase at the same time points, indicating simultaneously production in the fermentation (Fig. 4c). 5HTP containing supernatant was harvested by centrifugation and filtration. In a second step serotonin production was conducted by mixing fresh fermentation medium with the 5HTP supernatant (4:1 ratio), glucose concentration was adjusted to 30% and pH to 6.7. The mixed medium was inoculated with the strain BL21(DE3) *ΔtnaA* harboring plasmid pCOLAJ23-*TDC*. Cell growth stopped 24 h after inoculation (Fig. 4d). The consumption of 5HTP and serotonin production had a strong correlation (Fig. 4 e-f). Serotonin was continuously accumulated until 44 h when it reached a steady value. The maximum serotonin production was observed 52 h after inoculation, 154.3 ± 14.3 mg/L. The initial tryptophan concentration in the medium was 5.66 ± 0.61 g/L, after 52 h fermentation tryptophan decreased to 2.66 ± 0.54 g/L. We also detected 2.91 ± 0.46 g/L tryptamine.

## Discussion

We have assembled a biosynthetic pathway for the production of 5HTP and serotonin from glucose using a tryptophan production strain carrying an engineered hydroxylase (*Ct*AAAH-LC) and a decarboxylase (TDC) enzyme. Additionally, PCD and DHPR genes were incorporated into the *E. coli* strain to establish an artificial regeneration system for the endogenous cofactor tetrahydromonapterin (MH4), which was previously proved to work [20, 27].

An alternative pathway that leads to the production of serotonin via tryptamine, was also analyzed and discarded. In preliminary experiments, we compared the tryptophan hydroxylation activity of an *E. coli* with the *Ct*AAAH-F enzyme against a strain that contained a tryptamine 5-hydroxylase gene and its respective NADPH-cytochrome P450 reductase [28]. We found a higher hydroxylase activity in the first strain. Therefore, we lean toward the production of serotonin via 5HTP. Regarding the decarboxylation reaction, aromatic L-amino acid decarboxylases (AADC) are present in animals, insects and plants. Although their high homology, animal and insects AADC accept a broad range of aromatic amino acids on contrary to plant AADC which exhibits exclusive substrate specificity depending on the indole or phenol group [29]. Previous studies showed that *C. roseus* TDC can use both tryptophan and 5HTP as substrate [21, 22]. In consequence, we choose to produce serotonin via 5HTP, which is also an advance since 5HTP is a valuable compound for the pharmaceutical industry.

We compared two strategies for the expression of the enzymes responsible for the cofactor recycling. PCD and DHPR genes were incorporated via plasmid transformation or integrated into the genome of a tryptophan producer strain. In both cases, the P_trc_ promoter controls the transcription of the bicistronic mRNA, and the main differences lies in the number of copies per bacteria. The strain in which *PCD* and *DHPR* were integrated into the genome possess one copy of the genes, whereas there are around 15 to 20 copies in the strain transformed with pBbE1k-2. The low 5HTP production in the TrpD-Gi strains might be due to a low regeneration rate of the MH4 cofactor given single-copy of the genes present in the strain.

When expressing *Ct*AAAH-F in our strains, we observed a higher production of 5HTP when *Ct*AAAH-F was under control of the medium strength promoter P_J23110_. Often the expression of recombinant proteins in host cells requires a significant amount of resources causing overload in the metabolism of the host [30]. The comparison of pACJ23-*Ct*AAAH-F and pACTrc-*Ct*AAAH-F suggests that the mild promoter seems to benefit the production of 5HTP.

Tryptophan and 5HTP productions are uncoupled in the strain TrpD-Pl / pACJ23-*Ct*AAAH-F. The 5HTP production increased once the tryptophan production was stabilized (see Fig. 2c). We did protein directed evolution to select for enzyme variants with higher activity using a fluorescent sensor dependent on the intracellular tryptophan concentration. This sensor was originally developed by Fang et al., [31] to report the production of deoxyviolacein. Three mutants (F197 L / F197I / E219C) were identified with higher activities than the original *Ct*AAAH-F, and in a second iteration step we combined the F197 L and E219C mutations and generated the strain *Ct*AAAH-LC. When compared with *Ct*AAAH-F, tryptophan and 5HTP production occur simultaneously in the strain TrpD-PI / pACJ23-*Ct*AAAH-LC (Figs. 2d and 4b). Iterative site mutagenesis (ISM) has proven before to be a useful approach for the improvement of the enantioselectivity, substrate acceptance or thermostability of different enzymes [32, 33]. We used ISM to increase the activity; finally, an enzyme with a lower K_m_ and a higher reaction velocity was selected. We predicted a certain degree of volume and shape changes in the binding pocket which remains to be confirmed by structural determination. Nevertheless, modeling and docking analyses provide useful hints about conformational variations. It is reasonable to assume that the size of the binding pocket changes with the substitution of phenylalanine for leucine at the position 197 and glutamate for cysteine in the residue 219. This new conformation probably stabilizes the enzyme-substrate-cofactor complex, which is important for the tryptophan hydroxylase activity [20].

In a first attempt to produce serotonin from glucose, we incorporated TDC in the strain TrpD-Pl / pACJ23-*Ct*AAAH-LC. Tryptophan and 5HTP are natural substrates of *C. roseus* TDC; however, apparent K_m_ value for tryptophan is 0.075 mM, whereas the K_m_ for 5HTP is 1.30 mM [21]. This may explain why we detected a much higher concentration of tryptamine (3.03 ± 0.32 g/L) than serotonin (not detected) in the supernatant after fermentation. We decided to regulate the *TDC* expression using the P_araBAD_ promoter and after 60 h, 1.81 ± 0.29 g/L and 1.66 ± 0.27 g/L of tryptamine were detected in the induced and the non-induced fermentations. Although the *TDC* gene was induced 24 h after inoculation when OD was around 10, we speculate that the low serotonin productivity is due to an unwanted leaky expression from the arabinose-induced promoter in early stages, this may reduce the tryptophan pool in detriment of the 5HTP pathway; consequently, serotonin production is low.

Despite the benefits of single culture with a recombinant strain, we decided to perform the serotonin production in a two-stage system. In the first stage 5HTP was produced from glucose with the TrpD-Pl / pACJ23-*Ct*AAAH-LC strain, then cells were eliminated to prevent cross-contaminations of the second stage. In this last step, the harvested supernatant containing 5HTP and tryptophan was used to grow BL21(DE3) *ΔtnaA* harboring plasmid pCOLAJ23-*TDC*, both products were hydroxylated and serotonin as well as tryptamine were detected in the supernatant. This is the first report of serotonin production from a simple carbon source, final production achieved was 154.3 ± 14.3 mg/L using the stepwise culture approach. This method enables flexibility for independent optimization of each reaction, i.e. 5HTP conversion stopped after 44 h of fermentation, probably due to the accumulation of tryptamine as it is a competitive inhibitor of the decarboxylation reaction [21]. Still, in order to reach industrially relevant level, the selectivity of 5HTP decarboxylation should be improved in detriment of tryptamine production, reducing the formation of byproducts during the process.

## Conclusions

The heterologous expression of synthetic pathways for the production of high-value compounds provides a rapid and robust access to desired molecules. Often these compounds require multi-step reactions for their synthesis from simple carbon sources; along with the pathway enzyme inhibition and side-reactions may hinder the carbon flow and affect the final yield and productivity. Protein and metabolic engineering strategies are effective approaches used to circumvent these problems. Stepwise culture is an extra method that complements the system biology toolbox. In the present study, we used a two-stage fermentation to minimize an undesired side reaction. In the first fermentation, we produced 5HTP from glucose, and in the second step, we converted the produced 5HTP into serotonin. To our knowledge, this is the first report of microbial production of serotonin from glucose as a simple carbon source. The *E. coli* system developed for the production of 5HTP and serotonin represents a potentially useful platform for development aimed at the industrial production of tryptophan derivatives.

## Methods

### Bacterial strains and plasmids

Strains and plasmids used in this study are listed in Table 2. *E. coli* 10β (New England Biolabs, Frankfurt) was used for general cloning purposes. The strains used for the production of tryptophan metabolites were derivate from the *E. coli* strain S028 [14]. The tryptophan repressor (*TrpR*) gene was eliminated from strain S028 by homologous recombination using the approach previously described by Datsenko and Wanner, [34]: an overnight culture of the strain S028 carrying pKD46 was grown at 30 °C in 10 mL of LB media supplied with 1 mM arabinose until an OD of 0.6. Afterward, the culture was chilled for 10 min on ice; the cells were washed three times with a cold 10% glycerol solution and finally resuspended in 400 μL of 10% glycerol. 100 ng of linear DNA (FRT-kanamycin-FRT gene flanked by *TrpR* regions) was mixed with 200 μL of electrocompetent cells. After electroporation (0.2 cm cuvette, 2.5 kV, 25 μF and 200 Ω), cells were mixed with 1 mL SOC and incubated at 30 °C for 1 h before plating them on LB agar with appropriate antibiotics; from here strain TrpD was generated. During the *TrpR* elimination a kanamycin-resistance (*kan*) gene was incorporated as a positive marker for selection, the *kan* gene was eliminated: i) using plasmid pCP20 which expresses a FLP recombinase, that acts on the repeated FRT (FLP recognition target) sites. Afterward, this strain was transformed with plasmid pBbE1k-2.2 which carries human pterin-4 alpha-carbinolamine dehydratase (*PCD*) and dihydropteridine reductase (*DHPR*) genes, involved in the regeneration of the cofactor necessary for the hydroxylation reaction (Fig. 1). From here, strain TrpD-Pl was derived. ii) the *kan* gene was exchanged with the *PCD* and *DHPR* genes, following the homologous recombination method described before, to generate strain TrpD-Gi.

**Table 2:** List of strains and plasmids used for the production of 5-hydroxytryptophan and serotonin

	Characteristics	Source
Strains		
S028	W3110 ∆lacU169 gal490λCI857 ∆(cro-bioA) rpsL (StrR) ∆aroF∆aroG ∆mtr ∆tnaA ∆tnaB ∆aroH::PJ_23119-rpsL-tac_-(aroG_S180F_-serA_H344A/N364A_) P_trc_-trpE_S40F_D CBA	Chen and Zeng, [14]
TrpD-Pl	S028 *∆TrpR* carrying pBbE1k-2.2	
TrpD-Gi	S028 ∆TrpR::PDC DHPR	
10-β		New England Biolabs
BL21(DE3)		New England Biolabs
BL21(DE3) *∆tnaA*		This study
Plasmids^a^		
pACYCDuet-1		Novagen
p*Ct*AAAH-W192F	pACYCDuet-1; aromatic amino acid hydroxylase from *Cupriavidus taiwanensis –* W192F	Mora and Zeng [20]
pACPJ23	pACYCDuet-1.2, P_T7_ swapped with P_J23110_	This study
pACPtrc	pACYCDuet-1.2, P_T7_ swapped with P_trc_	This study
pACPJ23-*Ct*AAAH-F	pACPJ23; aromatic amino acid hydroxylase-W192F from *Cupriavidus taiwanensis*	This study
pACPtrc-*Ct*AAAH-F	pACPtrc; aromatic amino acid hydroxylase-W192F from *Cupriavidus taiwanensis*	This study
pBbE1k-2	pBbE1k; pterin-4 alpha-carbinolamine dehydratase (PCD) and dihydropteridine reductase (DHPR) from human	Satoh et al., [42]
pSentrp	Intracellular L-tryptophan sensor	Fang et al., [31]
PSentrp(-LVA)	pSentrp; GFP with protease signal	This study
pCOLADuet-	pCOLADuet-1; *GSTΔ37* tryptamine	Park et al.,[26]
* GSTΔ37T5H + TDC*	hydroxylase and tryptophan decarboxylase	
pCOLADuet-*TDC*	pCOLADuet-1; tryptophan decarboxylase	This study
pBAD/His		ThermoFisher Scientific
pBAD-TDC	pBAD/His; tryptophan decarboxylase	This study
pKD46	Red recombinase expression plasmid from phage λ, temperature sensitive plasmid	Datsenko and Wanner, [34]
pCP20	FLP recombinase, temperature sensitive plasmid	Datsenko and Wanner, [34]

Plasmids pKD46 and pCP20 were eliminated at 37 °C. Colonies with correct insertion were verified by PCR and DNA sequencing. The first step of 5HTP degradation in an *E. coli* BL21(DE3) strain disrupted by eliminating the tryptophanase gene (*tnaA*), BL21(DE3)*ΔtnaA* was generated using the same method described above; this strain was used for protein evolution, screening and bioconversion from 5HTP to serotonin.

Plasmids pACPJ23 and pACPtrc were generated from pACYCDuet-1. Briefly: pACYCDuet-1.2, other than the T7 protomer, was amplified by PCR with appropriate primers. The amplicon was circularized using In-Fusion HD Cloning Kit (Clonetech, Saint-Germain-en-Laye) and synthetic sequences containing the P_J23110_ (BBa_J23110, http://parts.igem.org/Main_Page) and P_trc_ [35] promoters, thus generating pACPJ23 and pACPtrc. Aromatic amino acid hydroxylase from *C. taiwanensis* (W192F) [20] was subcloned into the newly generated plasmids using the conventional digestion and ligation methods. Tryptophan decarboxylase gene plus vector was amplified from pCOLADuet-*GSTΔ37T5H + TDC* [26] and the P_J23110_ promoter was added by In-Fusion, creating pCOLAJ23-*TDC*. TDC was also inserted into the pBAD vector following the same procedures described above. The GFP protein associated with the tryptophan intracellular sensor (pSentrp developed by Fang et al., [31]) was modified by the addition of a short peptide sequence (-LVA) to the C-terminal end of the protein [36]. Plasmids in the text with the suffix “.2” refers to the original plasmid without the *LacI* gene, deleted by conventional PCR amplification followed by digestion and ligation.

### Semi-rational creation of library and screening of tryptophan consumers with a fluorescent sensor

**Fig. 5:**
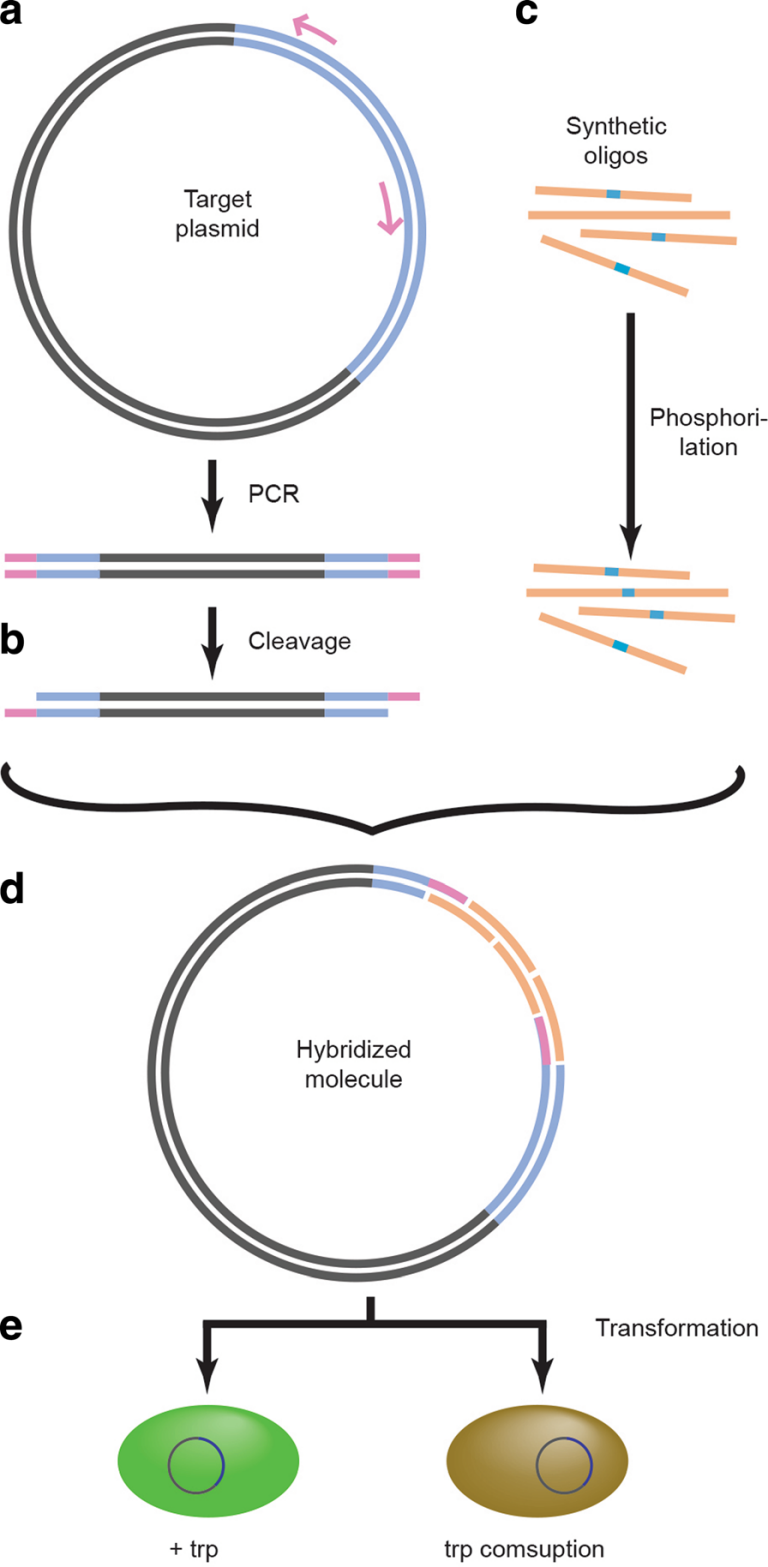
Library construction and screening strategies. **a** Plasmid and part of the *Ct*AAAH-F gene were amplified with phosphorothioate primers and (**b**) cleavage with I_2_/EtOH solution leaving sticky ends. The amplicon was hybridized with (**c**) synthetic phosphorylated oligos and (**d**) transformed into an *E. coli* strain which contains an intracellular tryptophan fluorescent sensor. **e** Tryptophan consumers can be distinguished by the lack or diminished formation of fluorescence

Two independent protein libraries were created using a site-saturated mutagenesis approach in the residues Phe197 and Glu219. These residues were selected due that they are in the binding pocket of the enzyme and play an important role defining the shape and volume near the aromatic ring of the substrate. The libraries were generated according to the following procedure: DNA with part of the *Ct*AAAH-F gene and the plasmid pACYCDuet-1.2 were amplified with phosphorothioate primers using Phusion High-Fidelity PCR Master Mix (ThermoFisher Scientific, Darmstadt). The fragment was treated with *Dpn*I enzyme and further diluted with 0.01 pmol/μL; cleavage was done according to Blanusa et al. [37]: 8.4 μL of PCR, 1 μL of buffer (0.5 M Tris-HCl, pH 9.0), 0.4 μL of iodine solution (100 mM iodine in ethanol), and 5 min incubation at 70 °C. In parallel, four oligonucleotides degenerated in the selected residues [24] were synthesized (one plus strand and one negative strand per site) and phosphorylated with a T4 Polynucleotide Kinase (ThermoFisher Scientific, Darmstadt). All oligos were designed in such a way that these would have sticky ends complementary to the cleavage fragment described above. DNA hybridization was achieved by mixing cleaved vector with 1 μL of the synthetic oligos (2.5 pmol/μL), to keep a ratio of 1:3 (vector: inserts). After 5 min at RT, the DNA complex was directly transformed into electrocompetent BL21(DE3)*ΔtnaA* cells generated by conventional methods (Sambrook and Russell, [38]). Cells were plated in LB-agar and grew overnight; afterward individual colonies were transferred to M9-agar plates supplied with 1 mM tryptophan and proper antibiotic. Colonies showing no fluorescence were transferred to an M9-agar-2 mM tryptophan plate. Successive 1 mM tryptophan steps were made until there was no evident change compared with the previous agar plate (Fig. 5).

Cells with less fluorescence were selected for DNA sequencing and best mutations were combined using QuickChange site-directed mutagenesis kit (Agilent, Santa Clara). Protein structures were predicted and analyzed described in [20]Mora-Villalobos and Zeng, (2017). Protein activity was evaluated according to [19]Kino et al. [19], and kinetic parameters were calculated using a Lineweaver-Burk plot.

### Production of 5HTP and serotonin by fermentation

Batch and fed-batch fermentations were done according to Chen and Zeng [14], with some modifications. Briefly, cells were cultured overnight in LB medium at 37 °C. The preculture was inoculated into 10 mL of seed medium with an initial OD_600_ = 0.1. The seed culture grew for 10 h at 30 °C; afterward, it was inoculated in 50 mL of fermentation medium to an initial OD_600_ = 0.1 and grown at 30 °C. When batch fermentations were done in shake flasks, 30 g/L of CaCO_3_ were added to the fermentation medium as a pH controller. Fed-batch fermentations were carried at 30 °C in 1.5 L jar fermenters (DASGIP, Jülich) with an initial volume of 500 mL. The pH was maintained at 6.7 by automatic addition of 25% NH_4_OH and 3 M H_3_PO_4_. The dissolved oxygen was set at 30% of air saturation varying in subsequent order the agitation speed, the oxygen content of the gas inlet and aeration rate. Glucose concentration was controlled by supplying a feeding solution with 60% glucose during the fermentation with a flexible feed rate.

The fermentation medium contained (per liter): glucose (30 g), MgSO_4_·7H_2_O (0.5 g), KH_2_PO_4_ (2 g), (NH_4_)_2_SO_4_ (4 g), yeast extract (1 g), monosodium citrate dihydrate (2 g), biotin (0.1 mg), DL-calcium pantothenate (0.5 mg), ascorbic acid (176 mg) and 10 mL of 100× stock trace elements. The stock trace elements solution was composed of (per liter in 0.1 N HCl) Na_2_MoO_4_·2H_2_O (2.5 g), AlCl_3_·6H_2_O (2.5 g), FeSO_4_·7H_2_O (10 g), CoCl_2_·6H_2_O (1.75 g), CaCl_2_·2H_2_O (10 g), ZnSO_4_·7H_2_O (0.5 g), CuCl_2_·2H_2_O (0.25 g), H_3_BO_3_ (0.125 g), Na_2_MoO_4_·2H_2_O (0.5 g). Seed medium was the same as the fermentation media except for 0.5 g/L instead of 5 g/L of MgSO_4_·7H_2_O. If necessary, the culture was harvested after the fermentation, the cells were separated by centrifugation and subsequent filtration. The supernatant was stored at − 80 °C until further use.

### Conversion of fermentatively produced 5HTP to serotonin

To produce serotonin an overnight culture of BL21(DE3)*ΔtnaA* strain harboring the plasmid pCOLADUET-*TDC* was inoculated in LB media; preculture and seed culture were prepared as described above. Because we had previously determined the proper mixture for cell growth, fermentation media and 5HTP-containing supernatant were mixed (4:1) and inoculated with the seed culture. Temperature (30 °C), pH (6.7) and dissolved oxygen (30%) were controlled and kept constant during the fermentation. Induction of tryptophan decarboxylase was carried out by the addition of 0.1 mM IPTG when OD_600_ was 10.

### Metabolites quantification

For fast tryptophan determinations, a spectrophotometric method [39] was used. 5HTP was quantified with a modified Gibbs assay [40]: 100 μL of 500 mM borate-NaOH buffer (pH 9) were mixed with 100 μL of the supernatant, followed by the addition of 4 μL of 0.5% (*w*/*v*) 2,6-dichloroquinone-4-chloroimide (Gibbs` reagent) in ethanol solution. After incubation at RT for 30 min, absorbance at 580 was measured for color product.

Aromatic amino acids, tryptamine, 5HTP and serotonin were analyzed using HPLC with a modified protocol described by da Luz et al. [41]. Proteins were precipitated by conventional TCA method; afterward, they were filtrated. Measurements were done on an Ultimate-3000 HPLC system (ThermoFisher, Darmstadt) with a binary gradient where eluent “A” was 140 mM sodium acetate with 0.1% *v*/v ACN and eluent “B” was 60% v/v ACN. The gradient was as follows: at 0 min 0% B; at 1 min 3% B; at 25 min 8% B; at 60 min 31.5% B; and remained constant from until 70 min at 100% B. All gradient changes were linear between the points given above. Separation was done using a Kinetex RP column (2.6 μm, C18, 100 × 4.6 mm, Phenomenex, Aschaffenburg) at 45 °C, and an injection volume of 10 μL was used.

## Additional file

Additional file 1: Supplementary data. (DOCX 308 kb)

## Abbreviations

5HTP: 5-Hydroxytryptophan
AAAH: Aromatic amino acid hydroxylase
GI: Genome integrated
PI: Plasmid integrated
T5H: Tryptamine 5-hydroxylase
TDC: Tryptophan decarboxylase
