# Supplementary material for: Synthetic pathways and processes for effective production of 5-hydroxytryptophan and serotonin from glucose in Escherichia coli
Source: J Biol Eng. 2018 Mar 15;12:3. doi: 10.1186/s13036-018-0094-7 (PMC5856393; doi:10.1186/s13036-018-0094-7)
Supplement: Supplementary file 1 — Supplementary data. (DOCX 308 kb) [file 13036_2018_94_MOESM1_ESM.docx]

**Supplementary Information**

**Synthetic pathways and processes for effective production of 5-hydroxytryptophan and serotonin from glucose in *Escherichia coli***

José-Aníbal Mora-Villalobos^1,2^ ([anibal.mora@tuhh.de](mailto:anibal.mora@tuhh.de)) and An-Ping Zeng^1§^ ([aze@tuhh.de](mailto:aze@tuhh.de))

^1^ Institute of Bioprocess and Biosystems Engineering, Hamburg University of Technology, Hamburg, Germany

^2^ Centro Nacional de Innovaciones Biotecnológicas, Centro Nacional de Alta Tecnología, San José, Costa Rica

^§^ Corresponding author


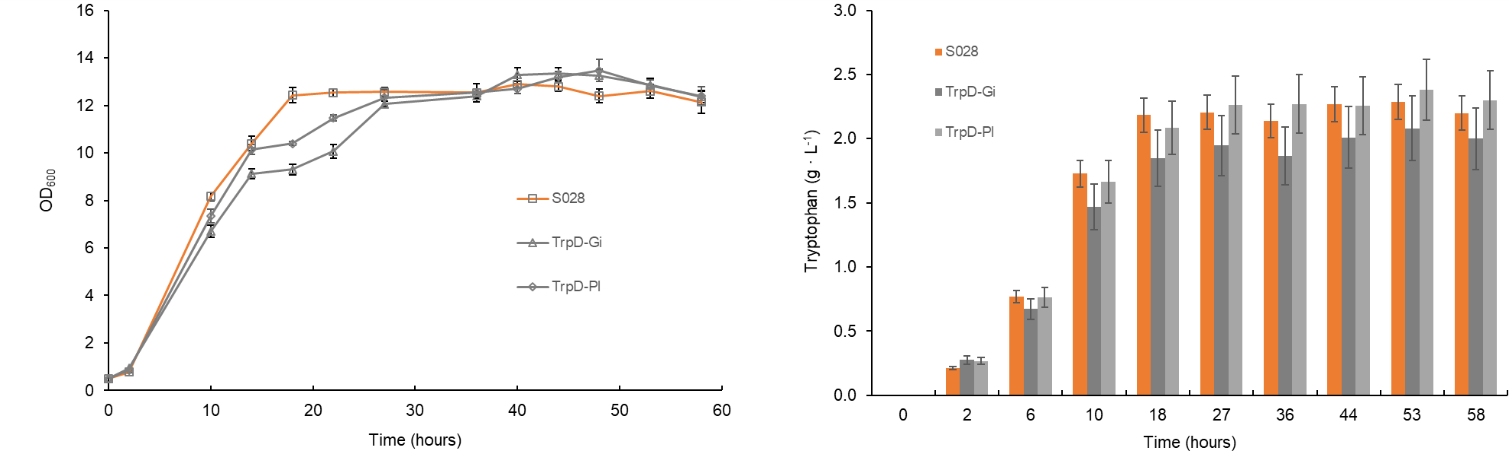


**Figure SI.1.** Cell growth and tryptophan production S028 and its derivatives strains for the production of 5HTP. Values in the graph are the average of triplicates, error bars correspond to the standard error of the mean (SEM).

**Table SI 1. List of primers used in this study.**

| **Primers** | **Sequence (5´ to 3´) ^a^** | **Purpose** |
| --- | --- | --- |
| CtAAAH_for | tatggatccatgtccatcgccatggccaccg | Gene cloning |
| CtAAAH_rev | atcaagcttcagatgtcttcggtatcggcc | Gene cloning |
| lacI_for | ctcgcatgcactctgcgacatcgtataacgttactggttt | Plasmid design |
| lacI_for | tatgcatgcgattcattaatgcagctggcacgaca | Plasmid design |
| P-J23110_rev | ctagtaggtttcctgtgtgactctagtagctagcattgtacctaggactgagctagccgtaaaatttcctaatgcaggagtcgcataag | Plasmid design |
| P-J23110-operator(lac)_rev | ggaattgtgagcggataacaattcccctgtagaaataattttgtttaactttaataaggagatataccatgggcagcagccatca | Plasmid design |
| P-trc_rev | atgaaggattatgtacatcaccatcatcaccatagccaggat | Plasmid design |
| P-trc-operator(lac)_rev | atgattaattgtcaaatttcctaatgcaggagtcgcataag | Plasmid design |
| trpR_for | cgctacaccagcggtaaggagatcc | Colony PCR |
| trpR_rev | aggctgttagatccacgcgtaatcgtc | Colony PCR |
| Cofactor1_for | atatgctatcgtactctttagcgagtacaaccgggggaggcattttgctttgtgtaggctggagctgcttc | Hom Rec |
| Cofactor2_for | gctaattcccatacggttctggcaaatattctgaaatgag | Hom Rec |
| Cofactor1_rev | ttgccagaaccgtatgggaattagccatggtccatatgaa | Hom Rec |
| Cofactor2_rev | attacgggtatttgtaggacggataaggcgttcacgccgcatccggcattcggtgcacgactagtagagagcgttcaccgacaaac | Hom Rec |
| araB_for | cgccacttcacgcatgttatcg | Colony PCR |
| araB_rev | cggtcggcagacaaattctcg | Colony PCR |
| araB-KO_for | Ttatagagtcgcaacggcctgggcagcctgtgccggggcggaagttggaagtgtaggctggagctgcttc | Hom Rec |
| araB-KO_for | atggcgattgcaattggcctcgattttggcagtgattctgtgcgagctttatgggaattagccatggtcc | Hom Rec |
| tio-Vector-Bottom | t*a*c*c*a*g*t*a*c*g*g*cgcgacagcatgt | Smart library |
| tio-Vector-Top | g*c*c*t*g*g*a*c*t*c*g*gccagcc | Smart library |
| Top Linker Fragment | cacggtcgagtttggcctgatccgcacggagcagggactgcgcatctacggcgccggcatcgtctcgagccagggggaatcgatctacagcctggactcg | Smart library |
| Fragment Phe197- AHN | gcgcagtccctgctccgtgcggatcaggcc**ahn**ctcgaccgtgtaccagtacagg | Smart library |
| Fragment Phe197- CDB | gcgcagtccctgctccgtgcggatcaggcc**cdb**ctcgaccgtgtaccagtacagg | Smart library |
| Fragment Phe197- CCA | gcgcagtccctgctccgtgcggatcaggcc**cca**ctcgaccgtgtaccagtacagg | Smart library |
| Fragment Glu219- AHN | tgtagatcga**ahn**cccctggctcgagacgatgccggcgccgtagat | Smart library |
| Fragment Glu219- CDB | tgtagatcga**cdb**cccctggctcgagacgatgccggcgccgtagat | Smart library |
| Fragment Glu219- CCA | tgtagatcga**cca**cccctggctcgagacgatgccggcgccgtagat | Smart library |

^a^ **n*** correspond to nucleotides in which sulfur substitutes for one of the oxygen in the phosphodiester bonds between the nucleotides.

**Gene sequence of *Cupriavidus taiwanensis* aromatic amino acid hydroxylase:**

ATGTCCATCGCCATGGCCACCGAAGCCCCCGGCGCCTTCCAGGGCACCCTGACCGACAAACTCAAGGAACAGTTCGACGCCGGCCTGCTGTCCGGCCAGGAACTGCGCCCGGACTTCACCATCGCGCAGCCGGTGCACCGCTACACCAGCATCGACCACGCCATCTGGCGCAAGCTATACGAACGCCAGGCCGAGATGCTGCGCGGCCGCGTCAGCGACGAGTTCCTGCAGGGGCTGGCCACGCTGGGCATGGAAAAAGACCGCGTGCCGGACTTCGACCAGCTCAACGAGACCCTGATGCGAGCCACCGGGTGGCAGGTCGTGGCCGTGCCCGGGCTGGTGCCGGACGAGGTCTTCTTCGAGCACCTGGCCAACCGCCGCTTCCCGGCCAGCTGGTGGATGCGCAAGCCCGAGCAGCTCGACTACCTGCAGGAGCCCGACTGCTTCCACGACGTGTTCGGCCATGTGCCGCTGCTGATCAACCCGGTCTTTGCCGACTACATGGAGGCCTACGGCAAGGGCGGCCTGAAGGCGGCCGGCCTGGGCGCGCTCGACATGCTGTCGCGCCTGTACTGGTACACGGTCGAGTTTGGCCTGATCCGCACGGAGCAGGGACTGCGCATCTACGGCGCCGGCATCGTCTCGAGCCAGGGGGAATCGATCTACAGCCTGGACTCGGCCAGCCCCAACCGGATCGGCTTCGATGTGCGCCGCATCATGCGCACGCGCTACCGCATCGACACGTTCCAGAAGACCTACTTCGTGATCGACAGCTTCGAGCAGCTGTTCGACGCCACCCGCCCGGACTTTGCGCCGCTGTACGAAGAACTGCGCGCGCAGCCGACGCTGGGCGCCGGCGACGTGGCGCCCGGCGACCAGGTGCTCAATGTCGGCACCCGCGAAGGCTGGGCCGATACCGAAGACATCTGA

**Tryptophan hydroxylation screening based on a tryprophan intracellular biosensor.**

Originally, the tryptophan intracellular biosensor designed and created by Fang et al. (2016), we modified it by adding a protease peptide signal fused to the C-terminal of the GFP to create an unstable variant of the protein (Andersen et al., 1998). The new variant of the biomolecular sensor (pSenTrpβ-GFP(LVA)), presented the linear response range between 0.5 and 1.8 mM. Also, cells harboring this plasmid presented a drastic reduction in the fluorescence after tryptophan was removed from the media and colonies harboring different *Ct*AAAH variants were clearly distinguishable in M9 plates supplied with tryptophan (Fig SI.2.).


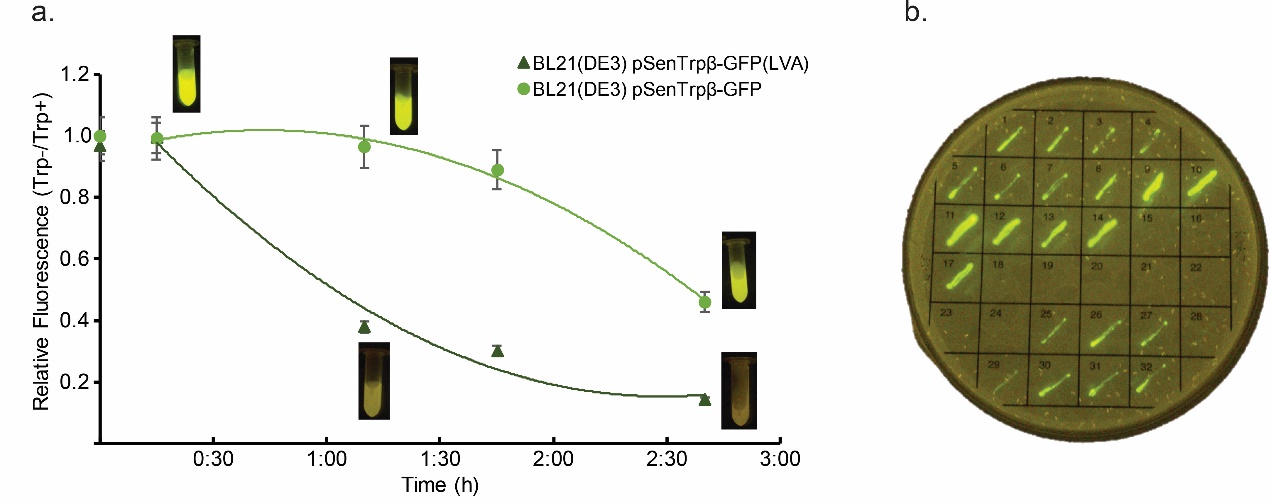


**Figure SI.2.** Performance of the tryptophan biomolecular sensor. (a) Flow cytometry measurements were done after cells were transferred in M9 media without tryptophan. (b) Fluorescence of cells harboring the pSenTrpβ-GFP(LVA) expressing different variants of CtAAAH (wild-type, W192F, Y224). Negative fluorescent control was also included.

Libraries, F197-Lib and Glu219-Lib, were plated in M9 media supplied with 1 mM tryptophan. Colonies with less fluorescence were transferred to fresh plates with 1 mM increase step of tryptophan until no change in fluorescence was evident (Fig SI.3.).


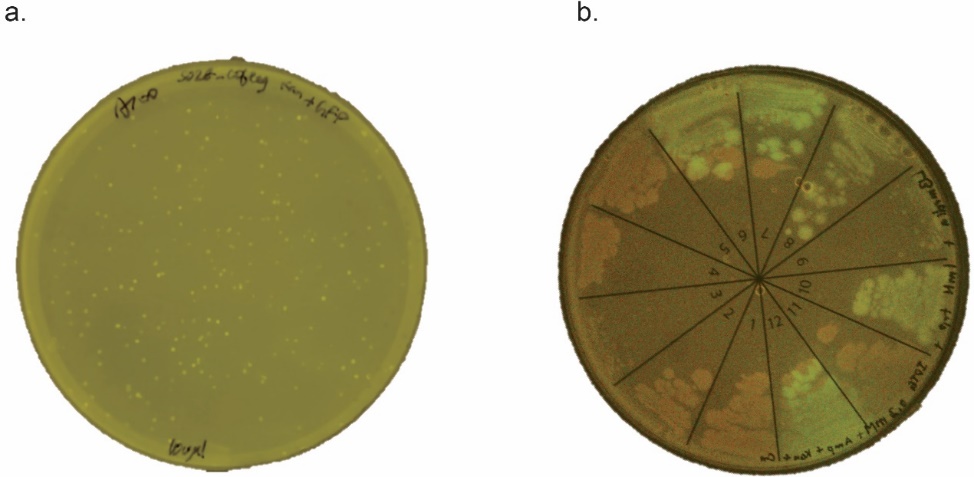


**Figure SI.3.** Screening procedure using pSenTrpβ-GFP(LVA). (a) Bacteria were plated in M9 plates supplied with 1 mM tryptophan, colonies with less fluorescence were transferred to a fresh M9 plate with 2 mM tryptophan. (b) Subsequent transferences with an increase in the tryptophan concentration were done until there was no distinguishable change in the fluorescent of the colonies when compared with the previous plate.
